# Prevalence of temporomandibular disorders and associated factors: a population-based study in southern Brazil

**DOI:** 10.1590/1807-3107bor-2025.vol39.092

**Published:** 2025-09-08

**Authors:** Alexandra Magalhães SILVEIRA, Graziela Oro CERICATO, Luiza Dal Zot von MEUSEL, Luiza Paloma dos Santos GIROTTO, Atais BACCHI, Yara Teresinha Corrêa SILVA-SOUSA

**Affiliations:** (a) Atitus Education, Graduate Program in Dentistry, Passo Fundo, RS, Brazil.; (b) Faculdade Paulo Picanço, School of Dentistry, Graduate Program in Dentistry, Fortaleza, CE, Brazil.; (c) Universidade de Ribeirão Preto – Unaerp, Graduate Program in Dentistry, Ribeirão Preto, SP, Brazil.

**Keywords:** Pain, Cross-Sectional Studies, Temporomandibular Joint Disorder, Epidemiology

## Abstract

The aim of this study was to assess the prevalence of temporomandibular disorder (TMD) and associated factors in an adult population in southern Brazil. The population-based sample (n = 4.65) included participants from Passo Fundo, a town in southern Brazil. The Fonseca Anamnestic Index was used to establish the prevalence of TMD. Sociodemographic and pathophysiologic factors and those that could cause tissue injury (trauma) were investigated. Data were analyzed using the chi-square or Fisher’s exact test and Poisson regression model (p < 0.05; 95%CI). The prevalence of TMD was 13.4%, and the most prevalent symptoms were pain in the neck and/or shoulders (30.1%), headache (26.0%), and presence of popping or clicking sounds (17.0%). None of the sociodemographic factors (sex, age, marital status, occupation, and education) were associated with the prevalence of TMD (p > 0.05). Among pathophysiologic factors, those associated with the prevalence of TMD were insomnia (PR: 1.83; 95%CI: 1.07–3.12), osteoporosis (PR: 2.50; 95%CI: 1.22–5.12), rheumatoid arthritis (PR: 1.99; 95%CI: 1.07–3.68), and xerostomia (PR: 1.36; 95%CI: 1.07–1.73). The factors that could cause tissue trauma/injury associated with TMD were sleep bruxism (PR: 2.16; 95%CI: 1.01–4.62), awake bruxism (PR: 2.44; 95%CI: 1.16–5.11), tongue pressure against the teeth (PR: 4.11; 95%CI: 1.95–8.65), and neck support of objects during work (PR: 2.94; 95%CI: 0.88–9.73). The prevalence of TMD was 13.44%, and it was associated with pathophysiologic factors and those that cause tissue trauma/injury, but not with sociodemographic factors.

## Introduction

Temporomandibular disorder (TMD) encompasses a group of musculoskeletal and neuromuscular conditions that involve the temporomandibular joints (TMJs), masticatory muscles, and all associated tissues, and it is the major cause of non-dental pain in the orofacial region.^1-3^ The most common TMD symptom is pain, which can occur spontaneously or upon contact with the masticatory muscles, TMJs, or the pre-auricular region, and it is normally aggravated by function. Symptoms and signs such as joint sounds and limitation or deviation of mandibular movements can also be found.^1^

No unequivocal universal cause of TMD has been identified. Based on observational studies, a multifactorial etiopathogenesis is considered. Factors that trigger TMD are called precipitating factors, those that increase the risk are considered predisposing factors, and those that interfere with the treatment or increase the progression of TMD are called perpetuating factors. It is important to note that, under different circumstances, the same factor, whether alone or in association, may fall into a different category.^1-3^ Therefore, a model of analysis based on a direct relationship between cause and effect is not suitable for this pathology.^1^ The most accepted etiological factors are tissue injuries caused by direct and indirect trauma or microtrauma; pathophysiologic factors, comprising systemic diseases and genetic predispositions; and behavioral psychosocial factors.^1-3^ Studies also suggest that there may be several unidentified factors that predispose individuals to the development of TMD.^3^

Population-based epidemiological studies are of interest for TMD. The prevalence of TMD reported in studies varies considerably due to its multifactorial etiology.^4^ In an attempt to standardize methodological approaches in epidemiological studies, several proposals have been made over the years. Questionnaires that assess signs and symptoms, such as the DC/TMD^2^ and Helkimo,^5,6^ have been introduced. The Fonseca Anamnestic Index,^7^ a more compact version for symptom assessment, has shown a 95% correlation with the Helkimo index. It consists of 10 questions, which are then transformed into an index. Moderate and severe scores indicate the need for treatment and/or further evaluation.^8^

Considering the importance of population-based epidemiological studies on TMD, the objective of this study was to assess the prevalence of TMD in the adult population of a town in southern Brazil and analyze the associated factors.

## Methods

This was a population-based cross-sectional study conducted with the adult population of a town in southern Brazil. The study was approved by the local Research Ethics Committee (process number: 2.225.924). Data were collected in 2018 by three previously calibrated researchers, with an interrater kappa value greater than 80%.

Sample size was calculated based on the latest census data for Passo Fundo, Rio Grande do Sul, Brazil, which revealed a population of 184,826 inhabitants.^9^ A 5% sampling error and 95% confidence interval were used. The sample size was increased by 20% to account for possible losses, resulting in a final sample of 465 individuals.

The 465 individuals were drawn proportionally to the number of residents in each of the 22 census sectors of the urban population. Each census sector consists of several neighborhoods; therefore, individuals were proportionally allocated to the neighborhoods within each sector. Households within each neighborhood were selected by systematic random sampling.^9^

The inclusion criteria were: male and female individuals aged 18 years or older residing in the sampled household at the time of data collection who signed the informed consent form. Exclusion criteria were: individuals who had experienced accidents, undergone facial surgery or radiation therapy in the past six months, or those with cognitive impairment.

The prevalence of TMD was determined according to the Fonseca Anamnestic Index,^7,10,11^ which consists of 10 questions with three possible answers: “yes,” “no,” or “sometimes.” The questions assess the following symptoms: (1) difficulty opening the mouth, (2) difficulty moving the jaw laterally, (3) discomfort or muscle pain when chewing, (4) headache, (5) neck and/or shoulder pain, (6) pain in or near the ear, (7) presence of joint sounds, (8) self-perception of normal bite, (9) self-perception of occlusion, particularly related to chewing on both sides, and (10) facial pain upon waking. Each “yes” answer was assigned a score of “2”, for “sometimes,” a score of “1,” and for “no,” a score of “0.” In questions 6 and 7, if bilateral symptoms were present, a score of “1” was added. In question 4, a score of “1” was assigned when pain was reported as frequent (more than twice a week) and/or intense. The scores were summed to classify the sample in relation to TMD, hereinafter referred to as the TMD index. Scores ranging from 0 to 3 were categorized as “no TMD”; 4 to 8 as “mild TMD”; 9 to 14 as “moderate TMD:” and 15 to 23 as “severe TMD.” Individuals with moderate or severe TMD indices were considered to have TMD, following the classification criteria of the Fonseca anamnestic index.

Data on sociodemographic factors were collected, including the following independent variables: age, sex, marital status, educational level, and occupational (work) status. The following pathophysiologic factors were assessed: smoking status, diabetes, insomnia or difficulty sleeping, joint problems, osteoporosis, xerostomia, and sadness or depression. Episodes or habits that could cause occlusal trauma or injury to other structures of the stomatognathic system were classified as “traumatic factors.” These factors included clenching the teeth during the day, grinding the teeth at night, pressing the tongue against the teeth, biting nails, chewing gum, working while holding something against the neck, and having been punched or hit in the face (direct trauma).

Data analysis was performed using Stata 12 software (StataCorp. LCC, College Station, USA). First, the main outcome was described by presenting the prevalence and severity of TMD and by subsequently presenting the prevalence of TMD symptoms. The association between the outcome (TMD) and the categorized exposure variables was analyzed using contingency tables, and the prevalence ratio (PR) was estimated. Bivariate analysis was performed to examine the association between the TMD outcome (binary: absent/present) and the exposure variables (numeric or categorical). The factors associated with TMD were subdivided into sociodemographic factors (age, sex, marital status, education, and occupation), pathophysiologic factors (diabetes, insomnia, arthritis, osteoporosis, xerostomia, smoking status, and sadness/depression), and factors causing tissue trauma/injury (microtrauma from parafunctions and direct trauma), as shown in Figure. Associations between the outcome and exposure factors were analyzed using Poisson regression models. Age and sex were included in the multivariate analysis, whereas variables with p ≤ 0.20 were included in the bivariate analysis. A significance level of 5% was set for all tests.

**Figure:**
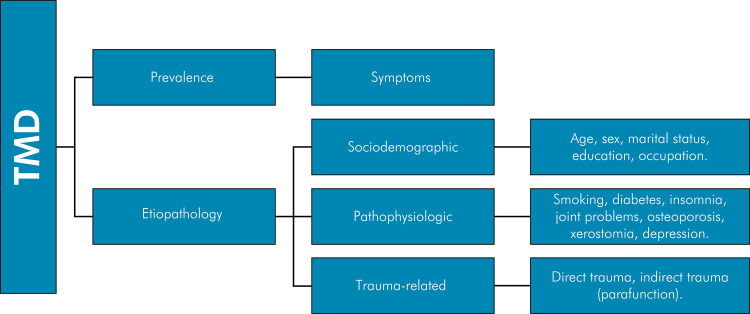
Experimental design of the study.

## Results

The study sample consisted of 465 individuals (136 males and 329 females) with a mean age of 43.6 years (range:18 to 96 years). The prevalence of TMD was 13.4%, with 10.4% (n = 48) classified as moderate and 3.0% (n = 14) as severe.

Table 1 presents the frequency of TMD symptoms reported according to the Fonseca Anamnestic Index. The most common symptoms were neck and/or shoulder pain (30.1%), headache (26.0%), and TMJ sounds (17.0%). Table 2 shows that none of the sociodemographic factors (sex, age, marital status, occupation, and education) were associated with the presence of TMD (p > 0.05).

**Table 1:** Prevalence of TMD symptoms (%) and total sum of points (considering individuals who answered ‘yes’ or ‘sometimes’) according to the scoring criteria of the Fonseca Anamnestic Index.

Items	Yes	No	Sometimes	Total Sum
1- Difficulty opening the mouth	6.9	88.0	5.1	88
2- Difficulty moving the jaw from side to side	5.1	87.0	7.9	84
3- Fatigue or muscle pain when chewing	11.8	76.8	11.4	163
4- Frequent headaches	26.0	55.1	18.9	330
5- Pain in neck and/or shoulders	30.1	48.6	21.3	379
6- Pain in or near the ear	11.6	78.3	10.1	155
7- TMJ sounds when opening the mouth or chewing	17.0	75.0	8.0	195
8- Feeling that teeth do not articulate well	28.6	67.5	3.9	284
9- Unilateral chewing	28.8	58.5	12.7	327
10- Facial pain when waking up in the morning	6.4	88.6	5.0	82

“yes” = 2 points, “sometimes” = 1 point and “no” value “0”. *Statistically significant p<0.05 for bivariate chi-square analysis.

**Table 2:** Association between the presence of TMD and sociodemographic factors.

Variables	Total (n = 465)	Without TMD		With TMD		PR (95%CI)^a^	p-value
		n	%	n	%		
Sex						1.81 (0.94–3.48)	0.05
Male	136	125	26.9	11	2.3		
Female	329	278	59.8	51	11.0		
Age (years)						1.00 (0.84–1.19)	0.20
18–30	119	108	23.2	11	2.4		
31–40	92	78	16.8	14	3.1		
41–50	87	68	14.6	19	4.0		
51–60	88	79	17.0	9	1.9		
> 60	79	70	15.1	9	1.9		
Marital status						0.98 (0.77–1.25)	0.73
Single	122	106	22.8	16	3.4		
Married	278	241	51.8	37	7.9		
Divorced	33	26	5.6	7	1.5		
Widowed	32	30	6.5	2	0.4		
Occupation						1.22 (0.78–1.91)	0.48
Never worked	33	30	6.5	3	0.7		
Worked, but not working now	138	120	25.8	18	3.9		
Currently working	294	253	54.4	41	8.8		
Education						0.89 (0.71–1.13)	0.28
No formal education	7	5	1.1	2	0.4		
Incomplete primary	37	30	6.5	7	1.5		
Incomplete elementary	67	59	12.7	8	1.7		
Incomplete high school	76	70	15.1	6	1.3		
Incomplete higher education	163	142	30.5	21	4.5		
Complete higher education	115	97	20.9	18	3.9		

^a^Multivariate Poisson regression, adjusted for sex and age and variables with p<0.20 in the bivariate analysis.

Table 3 shows the association of TMD with pathophysiologic factors, including some habits and systemic diseases. Conditions such as insomnia (PR: 1.83; 95%CI:1.07-3.12; p = 0.015), xerostomia (PR: 1.36; 95%CI:1.07–1.73; p = 0.0012), rheumatoid arthritis (PR: 1.99; 95%CI:1.07–3.68; p = 0.016), and osteoporosis (PR: 2.50; 95%CI:1.22–5.12; p = 0.005) showed a statistically significant association with the presence of TMD.

**Table 3:** Association between the presence of TMD and pathophysiologic factors (systemic diseases).

Variables	Total (n = 465)	Without TMD		With TMD		PR (95%CI)^*^	p-value
		n	%	n	%		
Diabetes							
No	421	369	79.4	52	11.2	0.43 (-0.36–1.22)	0.249
Yes	44	34	7.3	10	2.2		
Insomnia							
No	334	299	64.3	35	7.5	1.83 (1.07–3.12)**	0.015*
Yes	131	104	22.4	27	5.8		
Arthritis							
No	407	359	77.2	48	10.3	1.99 (1.07–3.68)**	0.016*
Yes	58	44	9.5	14	3.0		
Osteoporosis							
No	428	379	81.5	49	10.5	2.50 (1.22–5.12)**	0.005*
Yes	37	24	5.2	13	2.8		
Xerostomia							
No	341	302	65.0	39	8.4	1.36 (1.07–1.73)**	0.0012*
Yes	124	101	21.7	23	5.0		
Cigarette smoking							
Never smoked	318	279	60.0	39	8.4	0.99 (0.70–1.39)	0.43
Smoked, but quit	67	59	12.7	8	1.7		
Currently smoker	80	65	14.0	15	3.2		
Depression							
No	349	301	64.7	48	10.3	1.49 (0.86–2.58)	0.122
Yes	116	102	21.9	14	3.0		

^*^Multivariate Poisson regression, adjusted for sex, age, and variables with p < 0.20 in the bivariate analysis.**Statistically significant p < 0.05 for bivariate chi-square analysis; ***Statistically significant (p < 0.05) for multivariate analysis.

Table 4 presents the association between TMD and episodes or habits that could cause tissue trauma/injury, including microtrauma and direct trauma. The multivariate analysis revealed statistically significant associations (PR; 95%CI) between TMD and factors such as grinding teeth at night (sleep bruxism) (PR: 2.16; 95%CI:1.01–4.62; p = 0.020), clenching teeth during the day (awake bruxism) (PR: 2.44; 95%CI:1.16–5.11; p = 0.008), pressing the tongue against the teeth (PR: 4.11; 95%CI:1.95–8.65; p < 0.001), and holding something against the neck while working (PR: 2.94; 95%CI:0.88–9.73; p = 0.040).

**Table 4:** Association between the presence of TMD and trauma-related factors.

Variables	Without TMD	With TMD	PR (95%CI)^*^	p-value
	(%)	(%)		
Have you had any trauma to the face?				
No	69.3	9.9	1.15 (0.49–2.68)	0.72
Yes	17.4	3.4		
Do you work holding something against the neck?				
No	83.9	12.0	2.94 (0.88–9.73)	0.04**
Yes	2.8	1.3		
Do you grind your teeth at night?				
No	72.7	8.6	2.16 (1.01–4.62)***	0.02**
Yes	14.0	4.7		
Are you aware of clenching your teeth during the day?				
No	57.9	5.6	2.44 (1.16–5.11)***	0.008**
Yes	28.8	7.7		
Do you press the tongue against your teeth?				
No	75.3	7.3	4.11 (1.95–8.65)***	<0.001**
Yes	11.4	6.0		
Do you bite your nails, objects, or the lip?				
No	71.0	9.5	1.64 (0.72–3.72)	0.198
Yes	15.7	3.9		
Do you chew gum?				
No	77.2	10.8	1.84 (0.75–4.50)	0.143
Yes	9.5	2.6		

^8^Multivariate Poisson regression, adjusted for sex, age, and variables with p < 0.20 in the bivariate analysis.**Statistically significant p < 0.05 for bivariate chi-square analysis; ***Statistically significant (p < 0.05) for multivariate analysis.

## Discussion

The prevalence of TMD in the present study was 13.4%, which is in line with the outcomes reported in the literature. Agerberg and Inkapool^12^ found a prevalence of 12.5% of TMD in a population-based sample using the Helkimo questionnaire. Gillborg et al.^13^ obtained an 11.0% prevalence evaluating individuals aged between 20 and 89 years. Yekkalam and Wänman^4^ observed that 15% of individuals aged 35 to 75 years required TMD treatment. Melo et al.^14^ found a 14% prevalence using the RDC-TMD in a low-income population. Different prevalence rates were also reported in the literature, ranging from 1.6%^15^ to 27%.^16^ This variation in outcomes observed in epidemiological studies likely results from factors such as study design, of the methods used to evaluate signs and symptoms, and criteria used to establish the need for treatment.

Sociodemographic factors did not have a significant association with the prevalence of TMD in the present study. No statistically significant differences in the prevalence of TMD were noted between females and males. These data corroborate those found in previous population-based studies.^2,15,17^Other studies, however, found a higher prevalence of TMD among women,^18^ suggesting that this occurs either due to high levels of estrogen and polymorphic estrogen receptors in the temporomandibular joint, or due to a higher prevalence of depression and stress in the female population.^19^ Regarding age, some studies demonstrated a higher prevalence of TMD in individuals aged between approximately 30 and 50 years.^4,20^ Nevertheless, although the present study demonstrated a higher prevalence of TMD in individuals aged 41 to 50 years (21.8%), this difference was not statistically significant.

The lack of an association between TMD and marital status is consistent with other studies,^14,21,22^ except for Gillborg et al.,^13^ who observed that individuals living with a partner experienced less TMD than those who lived alone or in other family arrangements, and for the study of Kim et al.,^15^ who found that the prevalence of TMD was higher among single individuals than among married ones. Likewise, in the present study, there was no relationship between the presence of TMD and occupation. This finding is in agreement with previous studies.^14,21,22^ However, Gillborg et al.^13^ observed that individuals who were unemployed or in early retirement reported significantly more TMD than those who were working or studying. Sener and Akgunlu^23^ observed that marital status and occupation are associated with TMD only when these factors cause personal stress.

Similar to the findings of the present study, previous analyses have not observed a correlation between the prevalence of TMD and level of education.^13,15,21,23^ On the other hand, Kim et al.^15^ found a higher prevalence of TMD in individuals who attended high school or had not completed higher education. Wieckiewicz et al.^24^ concluded that the prevalence of TMD among university students would be higher due to their high emotional stress and excitability. As in the present study, Chung et al.^25^ did not find a statistically significant association between TMD and age, sex, education, and occupation.

The most frequently reported TMD symptom in the present study was neck and/or shoulder pain, consistent with the findings of Rokaya et al.^26^ “Having a headache frequently” was the second most common symptom. However, the relationship between headache and TMD is still widely discussed in the literature, considering that the trigeminal system is the pain modulator for both.^26^ Self-reported TMJ sounds were observed in 17.0% of individuals in the present study, a prevalence that slightly exceeds the range of 8.9%-15.8 reported in previous studies.^20^ Nonetheless, because the present research was solely based on symptoms, without clinical and complementary assessments, it is not clear whether these sounds were due to disc displacement with reduction or to crepitus - disc displacement without reduction.

Regarding pathophysiologic factors, the present study observed the prevalence of TMD and its association with some habits and systemic diseases. These factors were identified based on their potential influence on tissue-forming processes (e.g., diabetes), immunological metabolism (e.g., sleep disorders), or on their manifestations in the orofacial region (e.g., xerostomia, arthritis, and osteoporosis).

Diabetes mellitus is a disease that, among other things, interferes with both collagen synthesis and trigeminal pain (via neuralgia), hence its relevance to TMD studies.^27^ Previous studies^15,27^ have found a higher TMD prevalence in diabetic individuals than in the control group. In the present study, diabetes was not associated with the presence of TMD, possibly because it was self-reported, and many individuals could be unaware of the disease.

Sleep disorders are often associated with TMD, especially myofascial pain, which is related to poor sleep quality and chronic fatigue.^28^ Insomnia was associated with pain in the present study. Some explanations are that ‘pain and sleep’ can be influenced by neuroendocrine mechanisms and the function of the autonomic nervous system. Sleep disorders and nociceptive input significantly influence the increase in sympathetic muscle tone in chronic pain syndromes, which will, among other things, result in fatigue. As for depression, the present study did not find any association with the prevalence of TMD, thus corroborating the findings of Tournavitis et al.^29^

Rheumatoid arthritis is a chronic, inflammatory, systemic disease that manifests predominantly in synovial joints, such as the TMJ. In the present research, there was a positive association between the presence of TMD and self-reported rheumatism or rheumatoid arthritis. These outcomes are in agreement with a previous study,^30^ in which the highest prevalence of TMD occurred in patients with rheumatoid arthritis and osteoarthrosis.

In the present study, 26.7% of the individuals reported experiencing xerostomia, which was associated with TMD. A previous study^31^ has also found a higher prevalence of TMD symptoms, especially myofascial pain, in patients with Sjögren’s syndrome. Furthermore, it has also been demonstrated that patients with orofacial pain had less salivary flow and more complaints of xerostomia than those in the control group.^32^ The reasons for this association are not entirely clear, with some hypotheses suggesting that chronic pain may lead to sensitization of interneurons in the vegetative areas of the hypothalamus and in the pain pathways.^33^

Self-reported osteoporosis/osteopenia was positively associated with the presence of TMD in the present study. This association could be attributed to joint dysfunction caused by a compromised TMJ architecture and poor bone quality, as well as hormonal changes associated with osteoporosis. The increase in cortisol and decrease in estrogen levels, found in osteoporosis, are known to aggravate pain, by increasing nociceptive responses through the peripheral and central nervous systems.^34^

In the present study, no significant association was found between the presence of TMD and whether individuals were smokers, ex-smokers, or non-smokers. Other epidemiological studies have also demonstrated that tobacco would not be an independent risk factor for the development of TMD.^15,35^ Conversely, the smoking factor has been associated with other comorbidities, such as bruxism, sleep disorders, depression, and anxiety.^1,35^ This association can pose challenges in establishing a clear cause-and-effect relationship.

The present study did not find an association between TMD and direct trauma, which contrasts with previous studies that report a higher presence of TMD in patients who experienced direct facial trauma.^1,36^ Note that the ability to recall TMD symptoms associated with a traumatic event may be impaired by the event itself, thus hindering a causal relationship between the traumatic event and TMD. This consideration was also made by the American Academy of Orofacial Pain (AAOP).^1^ The studied factors “chewing gum” and “biting nails, biting objects, or the lip” were also not associated with TMD.

To assess sleep bruxism, which can potentially lead to microtrauma, individuals were asked whether they ground their teeth at night. Afterwards, the positive answers were associated with the questions from the Fonseca Anamnestic Index, “feeling pain in the face when waking up” and “having difficulty opening the mouth,” so that a diagnosis of probable sleep bruxism could be made. In this context, there was an association between sleep bruxism and TMD, which is in agreement with previous studies.^1,37,38^ “Clenching the teeth during the day” also referred to as awake bruxism, is another factor widely accepted as being associated with TMD.^37,39^ Such association was also observed in the present study.

Other parafunctional habits such as “pressing the tongue against the teeth” and “working while holding something against the neck” were associated with TMD in the present study. Keela et al.^38^ evaluated sleep and wakefulness habits of patients with painful TMD and found that “pressing the tongue against the teeth” was the strongest predictor of chronic painful TMD. The habits “chewing gum” and “biting nails and some objects” were not related to TMD. In any case, the exact role of parafunctional habits in TMDs is still not completely clear, given the complexity of the topic.^1^

It is important to highlight some limitations of the present study. Firstly, this was a cross-sectional study, which restricts the ability to identify risk factors for TMD. Furthermore, data were obtained through self-reports and questionnaires, without the inclusion of physical examination or complementary tests or exams, such as blood tests, imaging exams or others, to confirm or rule out the presence of pathologies. Data collection in TMD studies can be conducted either by combining clinical examination with an anamnestic questionnaire, such as the Helkimo questionnaire,^5,6,12,16^ or by applying an anamnestic questionnaire alone, such as the Fonseca questionnaire.^2,7,15^ Fonseca et al.^7^ obtained a 95% reliability between their questionnaire and Helkimo questionnaire,^5,6^ thus validating its use for epidemiological research on TMD.^8,26^The Fonseca Anamnestic Index was used in this population-based study because the participants were recruited and evaluated in their own homes and also because a more concise tool for TMD evaluation was required. Nevertheless, note that the DC/TMD is the standard tool for TMD diagnosis.^2^ Some findings of this study, such as the lack of correlation between TMD and sociodemographic factors, should be further investigated, as recent literature review has identified some potential associations between these factors.^40^

## Conclusions

The prevalence of TMD was 13.4%, and the most prevalent symptoms were neck and/or shoulder pain, headaches, and TMJ sounds. None of the socioeconomic factors were associated with the prevalence of TMD. Among pathophysiologic factors, insomnia, osteoporosis, rheumatoid arthritis, and xerostomia were associated with TMD. As for the factors that could lead to tissue trauma/injury, sleep bruxism, awake bruxism, tongue pressing against the teeth, and working while holding something against the neck were associated with the prevalence of TMD.

## Data Availability

The datasets generated during and/or analyzed during the current study are available from the corresponding author on reasonable request.
